# Ultrasonic-assisted electrodeposition of Cu-Sn-TiO_2_ nanocomposite coatings with enhanced antibacterial activity

**DOI:** 10.1016/j.ultsonch.2021.105593

**Published:** 2021-05-19

**Authors:** Dmitry S. Kharitonov, Aliaksandr A. Kasach, Denis S. Sergievich, Angelika Wrzesińska, Izabela Bobowska, Kazimierz Darowicki, Artur Zielinski, Jacek Ryl, Irina I. Kurilo

**Affiliations:** a Soft Matter Nanostructures Group, Jerzy Haber Institute of Catalysis and Surface Chemistry, Polish Academy of Sciences, PL 30-239 Krakow, Poland; b Research and Development Center of Technology for Industry, 00-120 Warsaw, Poland; c Department of Chemistry, Electrochemical Production Technology and Materials for Electronic Equipment, Belarusian State Technological University, 220006 Minsk, Belarus; d Department Biotechnology, Belarusian State Technological University, 220006 Minsk, Belarus; e Department of Molecular Physics, Lodz University of Technology, PL 90-924 Lodz, Poland; f Department of Electrochemistry, Corrosion and Materials Engineering, Gdansk University of Technology, PL 80-233 Gdansk, Poland; g Institute of Nanotechnology and Materials Engineering, Faculty of Applied Physics and Mathematics and Advanced Materials Center, Gdansk University of Technology, PL 80-233 Gdansk, Poland; h Department of Physical, Colloid and Analytical Chemistry, Belarusian State Technological University, 220006 Minsk, Belarus

**Keywords:** Nanocomposite coating, Cu–Sn–TiO_2_, Ultrasonic-assisted electrodeposition, Electrochemical properties, Antibacterial properties

## Abstract

Copper-based coatings are known for their high antibacterial activity. In this study, nanocomposite Cu–Sn–TiO_2_ coatings were obtained by electrodeposition from an oxalic acid bath additionally containing 4 g/dm^3^ TiO_2_ with mechanical and ultrasonic agitation. Ultrasound treatment was performed at 26 kHz frequency and 32 W/dm^3^ power. The influence of agitation mode and the current load on the inclusion and distribution of the TiO_2_ phase in the Cu–Sn metallic matrix were evaluated. Results indicated that ultrasonic agitation decreases agglomeration of TiO_2_ particles and allows for the deposition of dense Cu–Sn–TiO_2_ nanocomposites. It is shown that nanocomposite Cu–Sn–TiO_2_ coatings formed by ultrasonic-assisted electrodeposition exhibit excellent antimicrobial properties against *E. coli* bacteria.

## Highlights

• Cu–Sn–TiO_2_ nanocomposite coatings were electrodeposited under mechanical and ultrasonic agitation;
• Effect of TiO_2_ nanoparticles and current density on structural and antibacterial properties was investigated.
• Distribution of TiO_2_ in the coatings improved under ultrasonic agitation;
• Antibacterial activity of Cu–Sn–TiO_2_ coatings was enhanced by ultrasonic agitation.

## 1 Introduction

Electrodeposition is a simple, well-developed, and low-cost technique that is commonly used to obtain various metal and alloy coatings on an industrial scale [1], [2]. Recently, fast development of nanotechnology promoted extensive studies on advanced multifunctional coatings, which possess improved, and often even unique properties as compared with traditionally used metallic coatings [3], [4], [5], [6]. Electrodeposition allows for the controlled incorporation of the second phase particles into the metal matrix, thus such composite coatings are usually characterized by improved microhardness, functional properties, wear- and corrosion resistance, which extends their potential applications [7], [8], [9], [10], [11], [12]. In this regard, electrodeposition attracts more and more attention in the fabrication of novel types of alloys and nanocomposites [13], [14], [15], [16], [17], [18].

Cu–Sn alloys have the advantages of decorative appearance, high corrosion resistance, and low toxicity, which make them widely used as decorative, protective, and functional coatings [19], [20], [21], [22]. Moreover, Cu–Sn coatings can be used as a promising substitution of Ni coatings, which are known to cause allergies and dermatitis when their corrosion products are in direct contact with human skin [1], or being carcinogenic when inhaled [23]. Currently, the development of applications for Cu-based coatings resulted in their use as antibacterial coatings due to the beneficial effect of copper ions towards the destruction of surface biofilms [24], [25], [26], [27], [28]. Nowadays, the intrinsic antimicrobial functionality of copper-based coatings is especially important. Such decorative coatings deposited onto fomite high-touch surfaces, for example, handholds, door handles, etc., in public places can be effective in mitigation of the virus spreading, including the infamous SARS-CoV-2 [29].

An effective way to further enhance the antibacterial properties of copper-based alloys is the introduction of dispersed second-phase materials [30]. To date, several types of nanocomposite coatings, e.g. Cu–Sn–SiC [31], Cu–Sn–graphite–Al_2_O_3_ [32], and Cu–Sn–TiO_2_ [33] with enhanced mechanical and physico-chemical properties were successfully obtained based on Cu–Sn alloys. In this regard, the implementation of titanium dioxide as a second-phase material has many advantages, such as its chemical inertness, anti-wear, and photocatalytic activity in various environments. However, high ionic strength of the plating bath and low sedimentation stability of TiO_2_ nanoparticles in aqueous media are obvious obstacles for the incorporation of the second phase particles into a metal matrix during electrodeposition. The increase in particles concentration causes sedimentation instability of the suspension at high concentrations, while reduced conductivity of the solution may cause problems related to mass transport. Subsequently, this results in a very low (<0.5 wt% [34], [35]) fraction of TiO_2_ in the electrodeposited composites. Usually, mechanical agitation is used to improve the sedimentation stability of a suspension during the deposition of composites.

The use of sonoelectrochemical modes for the deposition of composite coatings showed high practical potential since ultrasound can promote deagglomeration of second phase particles in the electrolyte and, consequently, provide fine dispersion of particles in the metal matrix [8], [18], [36], [37], [38], [39], [40]. In liquid media, ultrasonic treatment generates the acoustic cavitation phenomenon, which decreases the thickness of the diffusion layer and improves the mass transport [18], [41]. The key properties of the deposited coatings are also affected by the parameters of ultrasound used in the electrodeposition process. The most common reported operation conditions for electrodeposition of composite coatings are ultrasound frequency of 20–42 kHz and nominal power from 1.2 to 40 W/dm^3^ [18]. Such treatment results in the broadening of the operating cathodic current density, reduced porosity of the metal matrix, improved mechanical properties, and an increase in the quantitative incorporation of the second phase [8], [40], [42], [43], [44], [45], [46]. The use of excessive ultrasonic power can negatively affect the size distribution of the second phase in a metal matrix [47].

Another aspect of Cu–Sn electrodeposition is the composition of the plating bath. Most methods used for deposition of these alloys are based on cyanide-containing solutions, which make the process environmentally dangerous [48]. Several alternatives, such as pyrophosphoric- [49], [50], methanesulfonic- [51], [52], and sulfuric-based [22], [53], as well as non-aqueous electrolytes [54] were proposed.

To our best knowledge, no detailed information on the ultrasonic-assisted deposition of Cu–Sn–TiO_2_ coatings and their antibacterial properties have been previously reported in the literature. In our previous works [42], [43], we showed that ultrasonic treatment of the oxalic acid bath with a power input of 32 W/dm^3^ allows for extending the operating current densities from 0.5 to 1.0 A/dm^2^ and promotes deposition of smooth semi-lustrous coatings. Further increase in ultrasound power input and current density resulted in a decrease of the cathode current efficiency and lower adhesion of coatings due to the intensive hydrogen evolution. In this study, we investigated the influence of ultrasound treatment on the electrodeposition of nanocomposite Cu–Sn–TiO_2_ coatings with enhanced antibacterial properties. The effect of ultrasound treatment and current load on microstructure, quantitative and qualitative composition, distribution of TiO_2_ particles, and antibacterial properties against *E. coli* bacteria was evaluated. In addition, a comparative study on the effect of the mechanical and ultrasound agitation on the properties of obtained nanocomposite coatings was performed.

## 2 Experimental

### 2.1 Electrolyte and samples preparation

Composition of the used electrolytes and parameters of Cu–Sn–TiO_2_ electrodeposition are listed in Table 1. The operating conditions were selected based on the literature review [18] and our previous experiments [42], [43]. Electrolytes were prepared using double distilled water and reagent grade chemicals received from Belreachim (Belarus). Commercially available titanium dioxide nanoparticles (Degussa P25 TiO_2_, particle size of 10–40 nm) were used as received (Fig. S1 in the Supplementary information). After mixing all components, pH of the electrolyte was adjusted by 0.1 M H_2_SO_4_ and controlled by a Titroline Easy autotitration system with ±0.1 accuracy. After each experiment, the composition of the plating bath was corrected based on the results of the chemical analysis. Copper (M0) plates served as anodes and cathodes. Before experiments, substrates were successively ground by SiC sandpaper up to P2000 grit (particle size 5–7 μm), decreased in the solution containing, g/dm^3^: 30 Na_2_CO_3_, 30 Na_3_PO_4_⋅12H_2_O, and 3 SINTANOL DC10; rinsed with distilled water, and activated in 0.1 M H_2_SO_4_ for 1 min. Finally, the surface was thoroughly washed with distilled water. The working area of the cathode was 4 cm^2^.

**Table 1:** Bath composition and operating parameters for deposition of Cu–Sn–TiO_2_ composite coatings.

Bath composition/g/dm^3^		Electrodeposition parameters	
CuSO_4_ · 5H_2_O	20	Cathodic current density / A/dm^2^	0.5–1.5
SnSO_4_	8	Bath pH	5 ± 0.1
(NH_4_)_2_C_2_O_4_	55	Temperature / ^o^C	25 ± 1
C_2_H_3_O_2_Na	20	Ultrasonic frequency / kHz	26
TiO_2_	4	Ultrasonic power input / W/dm^3^	32

### 2.2 Electrodeposition of coatings

**Fig. 1:**
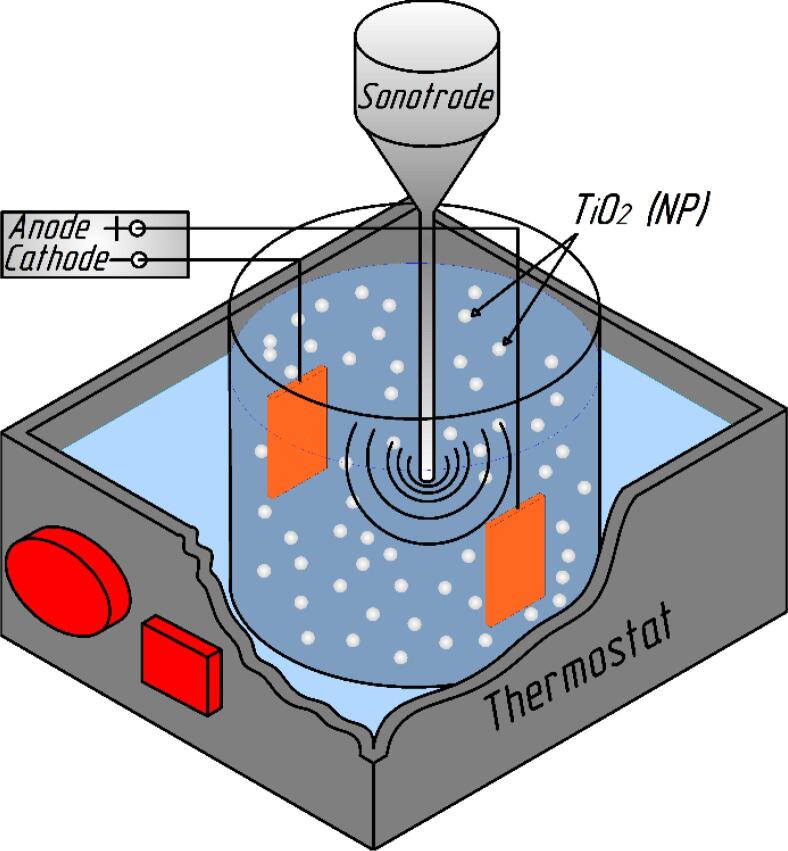
Schematic illustration of the experimental setup.

Electrodeposition was performed in an experimental setup consisting of a glass beaker with 0.3 dm^3^ of the plating solution, which was placed in the liquid thermostat as shown in Fig. 1. The ultrasonic treatment of the plating bath was provided by an UP200Ht ultrasound homogenizer equipped with a Hielscher submersible horn sonotrode of 12 mm in diameter.

The electrodeposition of Cu–Sn–TiO_2_ nanocomposite coatings was performed by a one-step process using a Mastech HY3005D-2 power supply. The temperature of the bath during electrodeposition was kept at 25 ± 1 °C by a WT3-1 thermostat. Coatings were obtained in the following agitation modes: without agitation (mode 0), mechanical agitation (mode 1), and ultrasound agitation (mode 2). In mode 2, the electrolysis was performed using the ultrasound frequency of 26 kHz and a power input of 32 W/dm^3^. The distance between the sonotrode and the cathode was 30 mm. In mode 1, parameters of the mechanical agitation (100 rpm) were adjusted experimentally to provide agitation conditions similar to those in the ultrasound regime. Prior to the electrodeposition in modes 1 and 2, the electrolyte was stirred mechanically or ultrasonically for 10 min. Reference Cu–Sn samples without TiO_2_ phase were deposited under the same conditions from the electrolyte without the second phase particles.

The thickness of all coatings deposited in the present work was 15 μm. After electrodeposition, samples were thoroughly rinsed with distilled water to remove remnant electrolyte and non-incorporated TiO_2_ particles from the surface of the samples.

### 2.3 Characterization of coatings

The X-ray diffraction patterns of the coatings were collected using an X’Pert PRO PANanalytical diffractometer operated at 40 kV and 30 mA by using Ni-filtered Cu K_α_ radiation. All patterns were recorded in the 2θ range of 35–80° at a scan rate of 2°/min. The crystallite size of the coatings was calculated using the Scherrer's equation:

(1) $$D=\frac{0.9\text{λ}}{\text{β}\cos\text{θ}},$$

where *D* is the mean size of the crystallite, 0.9 is the dimensionless shape factor, λ is the X-ray wavelength, β is the line broadening at half of the maximum intensity, θ is the Bragg angle.

The surface morphology and elemental composition of the deposits were examined using a HITACHI S-4700 scanning electron microscope (SEM) equipped with an EDX Thermo NORAN detector.

AFM measurements were conducted in the tapping mode using an NTEGRA Prima AFM system and NSG30 probes (NT-MDT, Russia) with a cantilever of nominal geometry (L × W × T) 125 ± 5 × 40 ± 5 × 4 µm and a tip radius of 10 nm. The measured areas contain 256 × 256 data points.

High-resolution XPS spectra were registered using a ThermoFisher Scientific Escalab 250Xi spectrometer, equipped with an Al Kα X-Ray source (spot size 250 μm) with the pass energy of 10 eV. Charge compensation was provided by calibration performed for adventitious carbon at the C1s peak (BE = 284.6 eV).

Polarization measurements were performed using an Autolab PGSTAT 302 N potentiostat/galvanostat at a linear sweep rate of 1 mV/s. A saturated silver/silver chloride electrode was used as the reference. All potentials reported in the paper are recalculated relative to the standard hydrogen electrode (SHE).

### 2.4 Antibacterial properties of coatings

Antibacterial properties of the coatings were examined according to the following procedure. Bare and Cu–Sn–TiO_2_-coated steel coupons were degreased in 99.9% ethanol to avoid any surface contamination and immediately immersed into a suspension of *E. coli* (ATCC 8739) bacteria. The incubation time was 1–2 h at 25 ± 1 °C and UV irradiation of 0.01 mW/cm^2^ intensity. Following the incubation time, samples were moved to darkness and rinsed with 0.01 dm^3^ of saline solution containing 0.01% of nonionic surfactant. The formed suspension was plated on BHI agar and the number of live bacteria was determined using the Koch method.

All the measurements reported in this study were triplicated unless otherwise stated.

## 3 Results and discussion

### 3.1 Ultrasound-assisted electrodeposition of Cu–Sn–TiO_2_ composite coatings

**Fig. 2:**
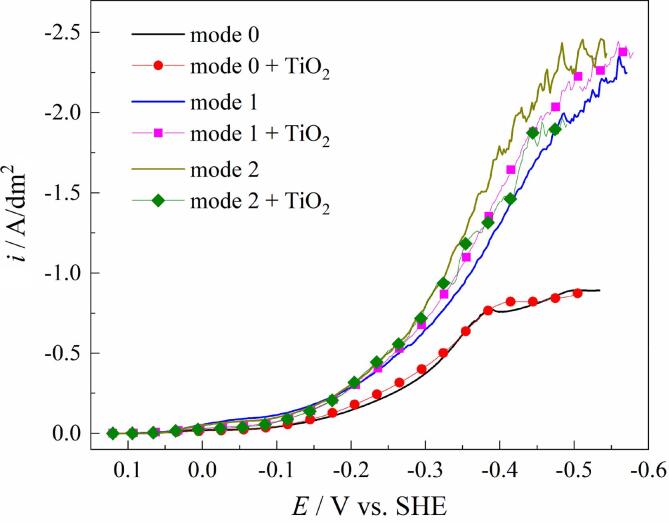
Cathodic voltammograms of copper electrode recorded in the studied electrolytes in quiescent conditions (mode 0) and under mechanical (mode 1) and ultrasonic (mode 2) agitation.

In order to investigate the effect of ultrasonic treatment on the processes of electrodeposition of Cu–Sn and Cu–Sn–TiO_2_ coatings they were also deposited under quiescent conditions (mode 0) and under mechanical agitation (mode 1). Fig. 2 shows cathodic voltammograms of the copper electrode recorded during electrodeposition of Cu–Sn and Cu–Sn–TiO_2_ alloys in the examined modes. In mode 0, the introduction of TiO_2_ in the deposition bath has a negligible effect on the cathodic sweep of the deposition curve. At potentials lower than –0.38 V electrodeposition proceeds at the diffusion limiting current density (*i*_L_) of 0.9 A/dm^2^. The plating bath was also characterized by low sedimentation stability. Visual inspection of the plating bath revealed that almost all introduced TiO_2_ was sedimented on the bottom of a beaker within 5–6 min of the deposition. The content of TiO_2_ particles examined by EDX, in this case was below the detection limit of the equipment used. It illustrates that Cu–Sn–TiO_2_ coatings cannot be effectively deposited under quiescent conditions. For this reason, only Cu–Sn coatings were deposited in mode 0 and further used as a reference. Agitation of the electrolyte (modes 1 and 2) improved stability and mass transfer in the plating bath and significantly affected electrodeposition kinetics. In these conditions, voltammograms were shifted to more positive potentials and did not have evident diffusion current plateau. Note that in the case of the ultrasound agitation (mode 2) polarization curves had a similar shape to those during mechanical agitation (mode 1). This indicates that the pre-selected parameters of the mechanical agitation (100 rpm) provided mass transfer in the near-electrode area comparable with that in the case of ultrasound agitation. Analysis of polarization curves showed that the agitation type affects voltammograms of Cu–Sn–TiO_2_ deposition in a different way. In mode 1, the addition of TiO_2_ particles shifted the voltammogram to more positive potentials at the same current load, providing a depolarization effect. Oppositely, the deposition curve in mode 2 shifted to more negative potentials, which can be caused by the interaction of ultrasound waves with the particles of the inert phase and a local decrease in intensity of the ultrasound due to acoustic phenomena [55].

### 3.2 Elemental, phase, and microstructural analysis of Cu–Sn–TiO_2_ composite coatings

Table 2 shows the quantitative and qualitative composition of the obtained coatings according to the EDX analysis. Despite the main components of the coatings, a small amount of C element, which was attributed to the surface contamination, was detected on the surface. In addition, the signal of O element could originate not only from TiO_2_ particles but also from the surface oxidation and contaminants. For these reasons, only Cu, Sn, and Ti elements were evaluated during the quantitative analysis. In quiescent conditions, Cu–Sn coating deposited at the cathodic current density of 0.5 A/dm^2^ contained 87.3 and 12.7 wt% of Cu and Sn, respectively. Mechanical and ultrasonic agitation provided favorable conditions for the deposition of the composite. The type of agitation used played an important role in the composition of the composite. Ultrasonic agitation resulted in the lower content of tin in the coating as compared to mechanical agitation at the same current load (4.7 and 5.1 wt%, respectively) due to the lower overpotentials of the electrodeposition process (Fig. 2). A noticeable decrease in the polarization (Fig. 2) resulted in a smaller current fraction consumed for the cathodic reduction of the electronegative component (Sn) of the metal matrix. Note that the content of TiO_2_ in the nanocomposite decreased too (0.4 and 0.3 wt% Ti in mode 1 and mode 2, respectively).

**Table 2:** Elemental composition of coatings deposited at different experimental conditions based on EDX analysis (scan area 50 × 50 µm^2^).

Agitation mode	Current density, A/dm^2^	Content in the coating, wt%		
		Cu	Sn	Ti
mode 0	0.5	87.3 ± 0.5	12.7 ± 0.5	–
mode 1	0.5	94.5 ± 0.4	5.1 ± 0.4	0.4 ± 0.1
	1.0	90.0 ± 0.5	9.5 ± 0.5	0.5 ± 0.1
mode 2	0.5	95.0 ± 0.4	4.7 ± 0.3	0.3 ± 0.1
	1.0	90.1 ± 0.2	9.6 ± 0.3	0.4 ± 0.1

With increasing of the current load to 1.0 A/dm^2^, the content of Sn in the resulting nanocomposite coatings increases to 9.5–9.6 wt% for mode 1 and 2, respectively, due to a higher cathodic polarization. The same was observed for Ti element, reaching the highest amount of 0.5 wt% in mode 1 (Table 2). However, the tendency of the lower Ti content in mode 2 was still obvious. Such dynamics of the TiO_2_ content in the Cu–Sn–TiO_2_ coating could be attributed to the cavitation phenomena, impeding the inclusion of larger agglomerates of the inert phase into the metallic matrix [1], [21].

**Fig. 3:**
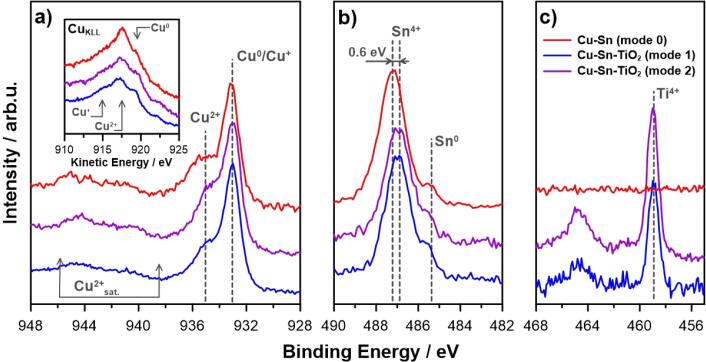
High-resolution XPS spectra of the samples deposited at cathodic current density of 0.5 A/dm^2^ for each studied agitation mode. The spectra reveal chemical composition in the binding energy range of Cu 2p_3/2_, with Auger Cu_KLL_ peak in the inset (a), Sn 3d_5/2_ (b), and Ti 2p (c).

The surface chemistry of the formed coatings will have a strong impact on their antibacterial activity. For this reason, a detailed XPS analysis was performed. High-resolution XPS spectra of Cu–Sn and Cu–Sn–TiO_2_ coatings obtained in various agitation modes at 0.5 A/dm^2^ are shown in Fig. 3. These spectra were measured in Cu 2p_3/2_, Sn 3d_5/2_, and Ti 2p binding energy ranges.

The Cu 2p spectra recorded on the surface of all investigated coatings show a complex multiplet structure. The Cu 2p_3/2_ peak for the most dominant component was located at 933.0 eV, which is characteristic of metallic Cu [56], [57]. However, it should be noted that Cu^+^ is also to be found within the same binding energy range. Therefore, a second scan was performed in the energy range of Auger Cu_KLL_ peak, which kinetic energy might be used to distinguish between various oxidation states of copper [58], [59]. Its results, which testify to the presence of a mixture of Cu^0^ and Cu^+^ species are to be found in the inset of Fig. 3a. Next, the positively shifted Cu 2p_3/2_ peak, located at approx. 935.1 eV, was recognized as Cu^2+^ in the form of copper hydroxides and/or carbonates formed on the surface most likely during post-electrolysis cleaning and the following exposure to ambient laboratory conditions [60]. Their presence was double-verified by the appearance of Cu^2+^ satellite features ranging from 940 to 945 eV and Auger Cu_KLL_ peak at 917.7 eV. When considering the copper chemistry, no significant differences were observed between each analyzed coating, as the Cu^2+^-to-(Cu^+^ or Cu^0^) ratio was typically found between 0.54:1 to 0.71:1.

Similar behavior was observed when analyzing Sn 3d spectra (Fig. 3b). The dominant tin compound on the surface of Cu–Sn coating was SnO_2_, verified by the Sn3d_5/2_ peak at 487.2 eV [61]. There is an observable shift in the SnO_2_ peak position of 0.6 eV between Cu–Sn coating and Cu–Sn–TiO_2_ coating. The negative binding energy shifts under cathodic polarization were previously observed and discussed for other electrode materials and connected with a lack of electronic equilibrium between the metal electrode and oxidized adsorbate species [62]. The latter, a significantly weaker component, originates from the metallic tin (Sn3d_5/2_ at 485.2 eV) [63]. It should be noted, that the total amount of tin at the coating surface differs between various samples, its share is nearly four times higher for Cu–Sn coating (38.4 at%) in comparison to Cu–Sn–TiO_2_ coatings (10.0 at% in case of mechanical agitation and 8.9 at% in case of ultrasonic agitation). The significantly higher Cu:Sn ratio for both Cu–Sn–TiO_2_ samples may suggest selective corrosion of tin. For Cu–Sn coatings with more than 10 wt% tin, corrosion occurs with formation of a uniform surface SnO_2_ thin film. At a lower Sn content, the surface is enriched in the corrosion products of both Cu and Sn [64].

Finally, the presence of TiO_2_ in the Cu–Sn–TiO_2_ samples was confirmed with the Ti 2p_3/2_ peak emerging at 459.2 eV [57], [65]. The peak position was not altered depending on the agitation mode used, however, the amount of titania at the surface was over three times higher in the case of ultrasonic agitation. Therefore, a conclusion may be drawn that ultrasonic agitation is a more effective approach for cathodic deposition of the composite coating. These results are opposite to the EDX observations (Table 2), where a slightly higher content of TiO_2_ was observed for mechanical agitation. That can be explained by the difference in the examined depth of these two methods. In the case of XPS, the penetration depth is only several nm, thus the outer surface composition was examined. Oppositely, EDX data show the elemental composition from several µm in-depth and is affected by the signal from the metal matrix to a large extent. The details of XPS analysis and peak deconvolution are summarized in Table 3.

**Table 3:** Surface chemical composition of coatings deposited at cathodic current density of 0.5 A/dm^2^ based on deconvolution results of high-resolution XPS spectra.

Coating	Composition, at%				
	Cu		Sn		Ti
	Cu^2+^	Cu^0^	Sn^4+^	Sn^0^	Ti^4+^
mode 0 (Cu–Sn)	25.9	35.7	36.9	1.5	–
mode 1 (Cu–Sn–TiO_2_)	29.1	53.3	9.4	0.6	7.6
mode 2 (Cu–Sn–TiO_2_)	25.4	42.2	7.6	1.4	23.4

**Fig. 4:**
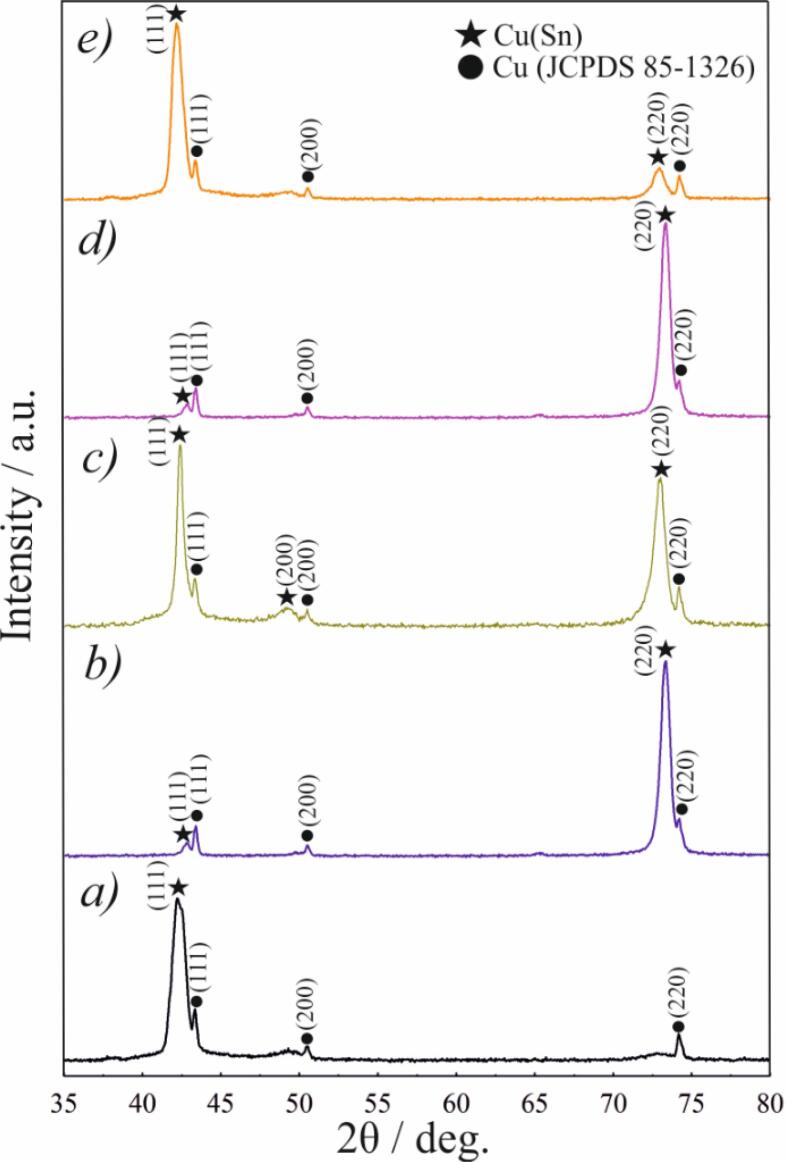
XRD patterns of coatings deposited in mode 0 (a), mode 1 (b, c), and mode 2 (d, e) at cathodic current density of 0.5 A/dm^2^ (a, b, and d) and 1.0 A/dm^2^ (c, e).

Fig. 4 shows XRD patterns of the coatings deposited in different agitation modes. The results show that the metallic matrix of obtained alloys is a single-phase substitutional solid solution of tin in copper [22], [66]. Due to a small thickness of the coating (ca. 15 µm), small peaks originating from the Cu substrate (JCPDS 85–1326) were also visible on the patterns. Calculated crystallite size based on Scherrer's equation and the positions of the main XRD peaks are summarized in Table 4. The latter are directly dependent on the content of Sn in the coating. The use of agitation changes the preferred orientation of the deposit from (1 1 1) to (2 2 0) plane at a current density of 0.5 A/dm^2^, which is due to the lower content of tin in the formed alloy. With an increase in current density, the preferred orientation of the deposit is (1 1 1) while XRD peaks are shifted to lower 2θ angles.

**Table 4:** Position of main XRD peaks and calculated crystallite size of the examined coatings.

Agitation mode	Current density, A/dm^2^	2θ, deg			*D*, nm		
		(1 1 1)	(2 0 0)	(2 2 0)	(1 1 1)	(2 0 0)	(2 2 0)
mode 0 (Cu–Sn)	0.5	42.20	–	–	12.70	–	–
mode 1 (Cu–Sn–TiO_2_)	0.5	42.40	–	73.32	23.90	–	30.14
	1.0	42.85	49.70	73.30	19.00	–	37.50
mode 2 (Cu–Sn–TiO_2_)	0.5	42.90	–	73.35	18.10	–	36.60
	1.0	42.15	–	72.95	13.40		30.00

Table 4 shows that the crystallite sizes were significantly affected by the agitation mode and in a less manner by the current density. The calculated size of the crystallites of coatings deposited in mode 0 at 0.5 A/dm^2^ was 12.70 nm. The size of the crystallites in the agitation modes 1 and 2 varied in the range of 13.40–37.50 nm depending on the orientation plane and was smaller for mode 2. As shown in Fig. 2, agitation in modes 1 and 2 decreases cathodic polarization. Smaller cathodic polarization decreases nucleation rate and, as a result, increases the size of the formed crystallites [67]. The increase in the cathodic current density from 0.5 to 1.0 A/dm^2^ increases cathodic polarization and decreases the size of the formed crystallites. Generally, applying of ultrasonic agitation during the electrodeposition slightly decreases the crystalline size due to the refinement in grain size [18]. However, a slight increase in the crystallite size of the coatings deposited in mode 1 and mode 2 can be also explained by the addition of TiO_2_. In this case, the second-phase particles may increase the number of structural defects, leading to an increase in the grain size [13], [68].

**Fig. 5:**
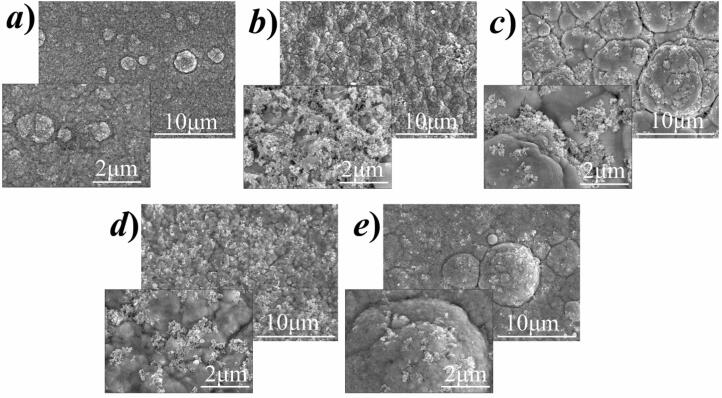
SEM images of coatings deposited in mode 0 (a), mode 1 (b, c), and mode 2 (d, e) at cathodic current density of 0.5 A/dm^2^ (a, b, and d) and 1.0 A/dm^2^ (c, e).

**Fig. 6:**
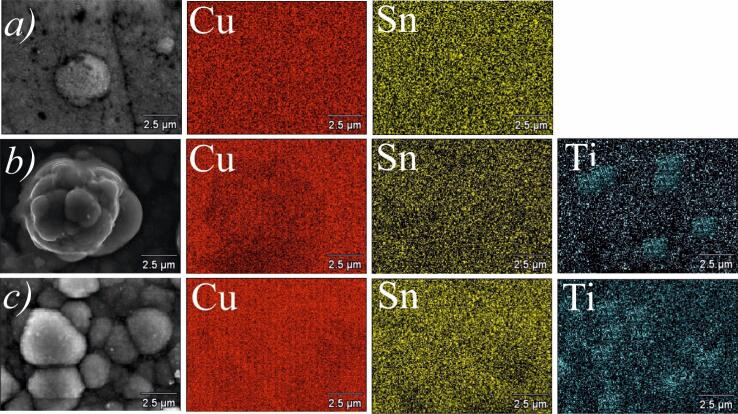
EDX elemental maps of coatings deposited in mode 0 (*a*), mode 1 (*b*), and mode 2 at cathodic current density of 1.0 A/dm^2^. Note that in mode 0 no signal from Ti element was recorded.

To study the influence of ultrasound treatment, the surface morphologies of the obtained coatings were examined by SEM and AFM. Fig. 5 shows the surface morphology of coatings deposited in the examined agitation modes at different current loads. It was observed that coatings deposited without agitation (mode 0) have a lot of globule-shaped granules on the surface (Fig. 5a). Agitation of the electrolyte affected the surface morphology. In mode 1, the surface was covered by numerous agglomerates, beneath those a dense coating is visible (Fig. 5b,c). The EDX analysis confirmed that these agglomerates are TiO_2_ nanoparticles, which were not embedded into the alloy matrix or covered by it (Fig. 6). Although agitation in mode 1 improved sedimentation stability of the electrolyte, it is evident that electrodeposition with mechanical agitation cannot evenly disperse TiO_2_ nanoparticles within the electrolyte, resulting in the formation of stacked agglomerates.

The Cu–Sn–TiO_2_ coatings deposited in mode 2 are characterized by higher homogeneity and smoother surface (Fig. 5d,e). The majority of the TiO_2_ particles were embedded into the Cu–Sn matrix. The introduction of ultrasound agitation improved the surface distribution of the TiO_2_ nanoparticles and impeded their aggregation, though it still could not be completely avoided.

To further support these observations, element distribution maps of the nanocomposite coatings (Fig. 6) were recorded by EDX. The data obtained clearly show even distribution of Cu and Sn in the structure of the metal matrix in all agitation modes. EDS maps also confirm that TiO_2_ nanoparticles were embedded in the Cu–Sn matrix during the electrodeposition process. Moreover, elemental maps in Fig. 6b clearly show that TiO_2_ nanoparticles form agglomerates up to several micrometers in size in the Cu–Sn–TiO_2_ coatings electrodeposited at 1.0 A/dm^2^ in mode 1. The highest concentration of TiO_2_ was detected in the surface regions corresponding to the spherical agglomerates. For coatings obtained under ultrasonic treatment in mode 2, the distribution of TiO_2_ particles over the surface is characterized by higher uniformity.

**Fig. 7:**
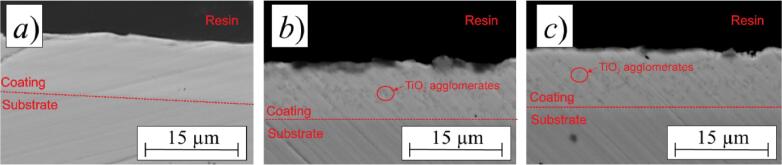
SEM cross-sectional morphology of Cu–Sn–TiO_2_ coatings deposited in mode 0 (*a*), mode 1 (*b*), and mode 2 at cathodic current density of 1.0 A/dm^2^.

In order to further analyze the distribution of TiO_2_ in the Cu–Sn matrix, the cross-sectional morphology of the coatings was examined by SEM as shown in Fig. 7. In modes 1 and 2, nanoparticles of the second phase were incorporated into the metal matrix throughout the whole thickness of the coatings without the presence of cracks or interconnected pores. The results clearly show that the coatings obtained in mode 2 are characterized by a smooth surface and a more uniform distribution of the TiO_2_ particles in the coating. The uniform distribution of the TiO_2_ particles in the metal matrix could enhance its antibacterial performance upon gradual degradation.

**Fig. 8:**
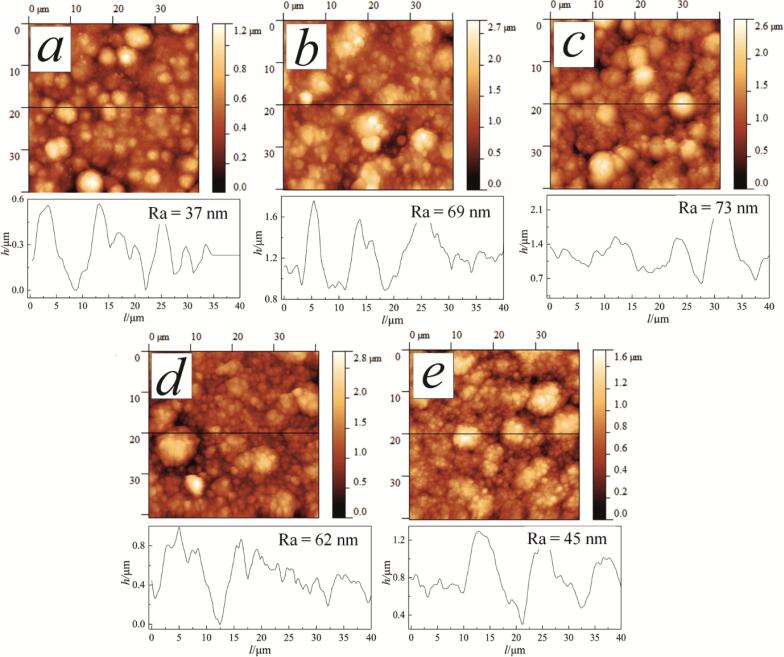
AFM topography images (scan area 40 × 40 µm^2^) of coatings deposited in mode 0 (a), mode 1 (b, c), and mode 2 (d, e) at cathodic current density of 0.5 A/dm^2^ (a, b, and d) and 1.0 A/dm^2^ (c, e). The line profiles show the topography variation along the lines in the corresponding maps.

Topography AFM images (surface area 40 × 40 μm) of the examined coatings are shown in Fig. 8. The average surface roughness, *R_a_*, of the coatings deposited in mode 0 was 37 nm. Agitation in modes 1 and 2 resulted in the introduction of TiO_2_ particles into the metal matrix and increased surface roughness due to their partial aggregation. The nanocomposites were made up of round-shaped granules with embedded nanoparticles. Comparison of the AFM images of the coatings obtained at the mechanical (mode 1, Fig. 8c) and the ultrasonic (mode 2, Fig. 8e) agitation showed that the size of grains is significantly lower in the latter case. The average surface roughness of the coatings deposited in mode 1 at 0.5 and 1.0 A/dm^2^ was 69 and 73 nm, respectively. The coatings deposited in agitation mode 2 have the average surface roughness of 62 and 45 nm, respectively, for the deposition current density of 0.5 and 1.0 A/dm^2^. This clearly shows that the ultrasonic agitation effectively reduced the surface roughness and improved its homogeneity.

### 3.3 Antibacterial properties of Cu–Sn–TiO_2_ composite coatings

**Fig. 9:**
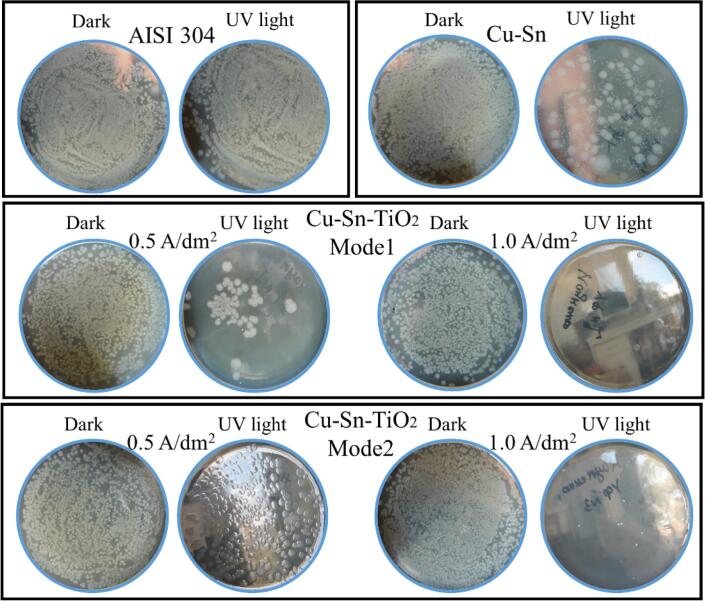
Optical photographs of *E. coli* colonies on BHI agar surface in Petri dishes after 20 min of bacterial tests.

The results of the antibacterial activity of the examined coatings are presented in Table 5 and Fig. 9. The concentration of active *E. coli* (ATCC 8739) bacteria in the initial suspension was 9.2 × 10^5^ CFU/mL. The results show that after 20 and 30 min of the experiment in dark conditions almost no inhibition of bacterial growth was observed for the coatings deposited in mode 0 and mode 1. In the case of the coatings deposited in mode 2 the concentration of active bacteria decreased by ca. 2 times. In the case of the experiment under UV illumination, significant inhibition of the bacterial growth from 9.2 × 10^5^ CFU/mL to (3.0–9.0) × 10^4^ CFU/mL (in 1.3–4.0 times) was observed already after 20 min of the experiment for the coatings obtained in mode 1 and 2. After 30 min, the concentration of the bacteria was below the detection limit (<10^4^ CFU/mL). Therefore, it can be concluded that Cu–Sn–TiO_2_ nanocomposites have high antibacterial activity towards *E. coli*. The best antibacterial performance showed the coatings obtained in mode 2. Their better antibacterial activity could be due to the higher surface content and more even distribution of the TiO_2_ particles over the surface of the coating. The mechanism of the antibacterial activity could be as follows [69], [70]: 1) rapid cell inactivation at the regulatory and signaling levels; 2) suppression of signaling pathways and the control of enzymatic activities; and 3) a strong decrease of the coenzyme-independent respiratory chains. These factors, together with the disintegration of the cell walls are the main reasons explaining the higher biocidal characteristics of Cu–Sn–TiO_2_ composite coatings. The results show that the use of Cu–Sn–TiO_2_ nanocomposites can effectively reduce the number of bacteria on the fomite high-touch surfaces in public places even without UV treatment.

**Table 5:** Antibacterial efficiency of Cu–Sn–TiO_2_ nanocomposite coatings towards *E. coli* ATCC 8739.

Agitation mode	Current density, A/dm^2^	Concentration of bacteria/ CFU/mL			
		Exposure under dark conditions for		Exposure under UV light for	
		20 min	30 min	20 min	30 min
Mode 0 (Cu–Sn)	0.5	(9.8 ± 1.2) × 10^5^	(8.6 ± 0.8) × 10^5^	(1.2 ± 0.3) × 10^5^	<10^4^
Mode 1 (Cu–Sn–TiO_2_)	0.5	(7.6 ± 0.8) × 10^5^	(8.8 ± 0.9) × 10^5^	(5.0 ± 0.4) × 10^4^	<10^4^
	1.0	(8.8 ± 0.7) × 10^5^	(7.2 ± 0.7) × 10^5^	(9.0 ± 0.8) × 10^4^	<10^4^
Mode 2 (Cu–Sn–TiO_2_)	0.5	(4.8 ± 0.6) × 10^5^	(4.2 ± 0.4) × 10^5^	(3.0 ± 0.4) × 10^4^	<10^4^
	1.0	(5.4 ± 0.5) × 10^5^	(5.0 ± 0.4) × 10^5^	(5.0 ± 0.4) × 10^4^	<10^4^
Stainless steel (control sample)	–	(9.6 ± 1.5) × 10^5^	(9.8 ± 1.1) × 10^5^	(5.2 ± 0.5) × 10^5^	(3.0 ± 0.5) × 10^5^

### 3.4 Mechanism of the formation Cu–Sn–TiO_2_ composite coatings

**Fig. 10:**
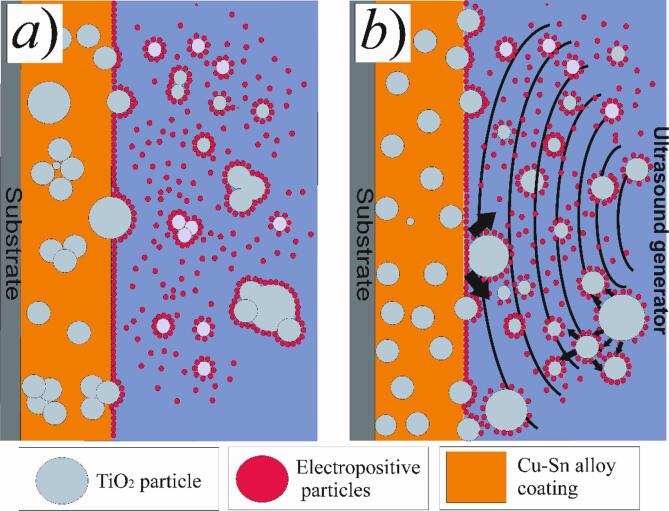
Schematic illustration of the mechanism of Cu–Sn–TiO_2_ coating deposition in the case of mechanical (a) and ultrasonic agitation (b) during electrolysis.

Based on the obtained results, the following mechanism of the electrodeposition of Cu–Sn–TiO_2_ coatings depending on the agitation mode can be proposed [39], [44], [71]. The sedimentation stability of TiO_2_ particles in water-based solutions is very weak [8]. For this reason, in the present study we implemented agitation to improve sedimentation stability during electrodeposition. In the case of mechanical agitation, as the current is applied to the electrolyte, copper and stannous ions are reduced on the substrate surface forming the initial layer of the metallic matrix (Fig. 10a). At the same time, metal cations can adsorb onto nanosized TiO_2_ particles, which are later migrating to the electrode surface under the electric field. Such TiO_2_ particles then diffuse through the double electric layer and adsorb on the surface of the cathode under the electric field. Note that such adsorption is rather weak and TiO_2_ particles at this stage can be easily removed from the surface by, for example, thorough rinsing. Finally, copper and stannous ions adsorbed on the TiO_2_ nanoparticle are reduced on the surface of the cathode, resulting in TiO_2_ particles embedded in the metal matrix.

The best results showed ultrasonic agitation, at which the depositing coatings were characterized by a fine distribution of TiO_2_ nanoparticles (Fig. 10b). Our results showed good agreement with this model. The uniform distribution of TiO_2_ nanoparticles in the coating also enhances its antibacterial performances.

## 4 Conclusions

In this work, Cu–Sn–TiO_2_ nanocomposite coatings were prepared by electrodeposition from an oxalic acid bath containing 4 g/dm^3^ TiO_2_ under different agitation regimes. The effect of the current load and ultrasound input of 32 W/dm^3^ on the structural and antibacterial properties of Cu–Sn–TiO_2_ nanocomposite coating has been evaluated. It was found that the agitation mode and current density affect the morphology and composition of the nanocomposites. Ultrasonic-assisted electrodeposition resulted in the significant improvement of the surface morphology and distribution of the TiO_2_ particles over the surface. The average roughness of the coatings was 69–73 nm and 45–62 nm for the mechanical and ultrasonic agitation modes, respectively. The coatings obtained by the ultrasound-assisted method are characterized by the highest antibacterial activity against *E. coli* bacteria.

## CRediT authorship contribution statement

**Dmitry S. Kharitonov:** Conceptualization, Investigation, Data curation, Formal analysis, Writing - original draft. **Aliaksandr A. Kasach:** Conceptualization, Methodology, Investigation, Visualization, Formal analysis, Writing - original draft. **Denis S. Sergievich:** Investigation. **Angelika Wrzesińska:** Investigation. **Izabela Bobowska:** Investigation. **Kazimierz Darowicki:** Resources. **Artur Zielinski:** Investigation. **Jacek Ryl:** Investigation, Formal analysis, Writing - review & editing. **Irina I. Kurilo:** Project administration, Supervision, Funding acquisition, Writing - review & editing.

## Declaration of Competing Interest

The authors declare that they have no known competing financial interests or personal relationships that could have appeared to influence the work reported in this paper.
