# Supplementary material for: Ultrasonic-assisted electrodeposition of Cu-Sn-TiO2 nanocomposite coatings with enhanced antibacterial activity
Source: Ultrason Sonochem. 2021 May 19;75:105593. doi: 10.1016/j.ultsonch.2021.105593 (PMC8233381; doi:10.1016/j.ultsonch.2021.105593)
Supplement: Supplementary data 1 [file mmc1.docx]

Supporting Information

**Ultrasonic-assisted Electrodeposition of Cu-Sn-TiO2 Nanocomposite Coatings with Enhanced Antibacterial Activity**

Dmitry S. Kharitonov^a,b^, Aliaksandr A. Kasach^c^, Denis S. Sergievich^c^, Angelika Wrzesińska^d^, Izabela Bobowska^d^, Kazimierz Darowicki^e^, Artur Zielinski^e^, Jacek Ryl^e^, and Irina I. Kurilo^c^

^a^Jerzy Haber Institute of Catalysis and Surface Chemistry, Polish Academy of Sciences, 30-239 Krakow, Poland

^b^Research and Development Center of Technology for Industry, PL–00120 Warsaw, Poland

^c^Belarusian State Technological University, 220006 Minsk, Belarus

^d^Lodz University of Technology, 90-924 Lodz, Poland

^e^Gdansk University of Technology, 80-233 Gdansk, Poland


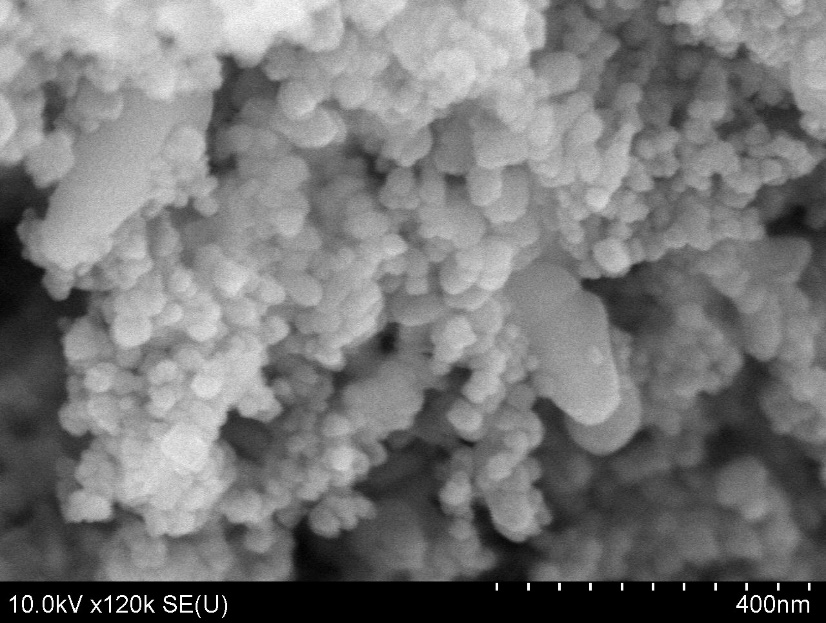


Fig. S1. SEM image of Degussa P25 TiO_2_ nanoparticles used in this work.
